# Longitudinal assessment of established risk stratification models in patients with monoclonal gammopathy of undetermined significance

**DOI:** 10.1038/s41408-024-01126-3

**Published:** 2024-08-27

**Authors:** Kosima Zuern, Thomas Hielscher, Annika Werly, Iris Breitkreutz, Sandra Sauer, Marc S. Raab, Carsten Müller-Tidow, Hartmut Goldschmidt, Elias K. Mai

**Affiliations:** 1 Heidelberg Myeloma Center, Internal Medicine V, Hematology, Oncology and Rheumatology, Heidelberg University Hospital, Heidelberg, Germany; 2 Division of Biostatistics, German Cancer Research Center (DKFZ), Heidelberg, Germany

**Keywords:** Myeloma, Risk factors, Disease-free survival, Myeloma

## Abstract

Risk of progression of monoclonal gammopathy of undetermined significance (MGUS) into multiple myeloma and related plasma cell disorders can be determined by three major risk stratification models, namely Mayo2005, Sweden2014, and NCI2019. This retrospective study of 427 patients with MGUS diagnosed according to the 2014 International Myeloma Working Group criteria aimed to describe and analyze the longitudinal applicability of these risk models. In all three models, the majority of patients remained at their baseline risk group, whereas small numbers of patients migrated to a different risk group. Proportions of patients among risk groups remained stable over time (e.g. Mayo2005 model, low-risk group, at baseline: 43%, after 1, 2, 3, 4, 5, and 8 years: 40%, 37%, 37%, 43%, 44%, and 43%). All three risk models reliably distinguished risk of progression at baseline, upon yearly reassessment (e.g. 1 year from diagnosis) and in time-dependent analyses. Upstaging to a high-risk category was associated with an increased risk of progression in all three models (Mayo2005: hazard ratio [HR] = 5.43, 95% confidence interval [95% CI] 1.21–24.39, *p* = 0.027; Sweden2014: HR = 13.02, 95% CI 5.25–32.28, *p* < 0.001; NCI2019: HR = 5.85, 95% CI 2.49–13.74, *p* < 0.001). Our study shows that MGUS risk stratification models can be applied longitudinally to repeatedly determine and improve individual risk of progression. Patient migration to higher risk categories during follow up should prompt more frequent monitoring in clinical routine.

## Introduction

Monoclonal gammopathy of undetermined significance (MGUS) is a precursor of multiple myeloma (MM) [1, 2]. It is typically detected incidentally during laboratory investigations and around 2.4% of the general population is affected by MGUS [3]. The current standard-of-care for MGUS is active surveillance with time intervals depending on the actual risk of progression [4]. A subset of patients with MGUS progresses to MM or related disorders with approximately 1% per year [5, 6].

Risk of progression of MGUS can be determined by three established models: Mayo2005 [7], Sweden2014 [8], and NCI2019 [9]. These models comprise clinical variables immunoglobulin (Ig) subtype, monoclonal protein (MCP) concentration, involved/uninvolved serum free light chain ratio (sFLCr), and immunoparesis of uninvolved Igs. These models reliably distinguish risk of progression of MGUS to MM and related plasma cell disorders requiring therapy at first diagnosis but have mostly not been assessed regarding their prognostic utility during follow up. Though longitudinal models for risk assessment such as the PANGEA model are available [10], the Mayo2005, Sweden2014 and NCI2019 are commonly applied in clinical routine since they have been developed based on cohorts with a long follow up (up to 30 years) and without active treatment.

To date, no studies have evaluated the longitudinal assessment of the major risk stratification models for MGUS diagnosed in accordance with the recent 2014 International Myeloma Working Group (IMWG) criteria [11]. A better understanding of individual disease dynamics and improved prediction of the risk of progression are therefore the basis for enhanced clinical monitoring and assessment over the course of the disease. This highlights the need to evaluate whether the established risk models work reliably at any timepoint in an individual course of disease and whether longitudinal application improves risk stratification.

Thus, our current study aimed to describe and analyze the longitudinal applicability of the major established risk models for MGUS in a contemporary cohort.

## Patients and methods

### Study cohort

This retrospective study included patients presenting with MGUS between 2005 and 2023 at Heidelberg University Hospital (Heidelberg, Germany). Follow up of the present cohort was current as of April 2024. The study was conducted according to the Declaration of Helsinki, and was approved by the ethics committee of the University of Heidelberg (S-597/2022 and S-578/2023). All patients gave written informed consent to participate in the study.

### Definitions, assessments and objectives

MGUS diagnosis was defined according to the current IMWG 2014 diagnostic criteria [11]. Briefly, MGUS is defined by serum MCP < 30 g/l, clonal bone marrow plasma cells (BMPC) < 10%, and absence of biomarkers of malignancy, end-organ damage, or light-chain amyloidosis. For light-chain MGUS, additional detection of an increased level of the involved light chain and an abnormal sFLCr and a urinary monoclonal protein < 500 mg/24 h are required. In line with previous analyzes [7, 9], this study also included patients with an MGUS diagnosis who did not receive imaging and/or bone marrow diagnostics at initial diagnosis, if there was no clinical evidence of MM.

Contemporary risk stratification models applied for this analysis comprised the Mayo2005 [7], Sweden2014 [8] and NCI2019 [9] models. The Mayo2005 model includes three risk factors: MCP ( ≥ 15 g/l; yes vs. no), abnormal sFLCr ( < 0.26 or > 1.65; yes vs. no) and Ig subtype (non-IgG vs. IgG). Based on the number of risk factors, patients were stratified into four risk groups: no risk factors (low), one risk factor (low-intermediate), two risk factors (high-intermediate), three risk factors (high). The Sweden2014 model added immunoparesis (yes vs. no) to the Mayo2005 criteria. Immunoparesis was defined as suppression of at least one uninvolved Ig isotype. Based on the number of risk factors the patients were assigned to five different risk categories (0–4 risk factors). The NCI2019 model excludes IgM MGUS and comprises four risk components: MCP ( ≥ 15 g/l; yes vs. no), abnormal sFLCr, ( < 0.1 or >10; yes vs. no), Ig isotype (IgA vs. other) and immunoparesis. In patients with light chain MGUS, the NCI2019 model comprised only abnormal sFLCr and immunoparesis as risk factors. MCP, Ig subtype, abnormal sFLCr and suppression of one uninvolved Ig accounted for one point, whereas suppression of two/three uninvolved Ig counted as two/three points, with three uninvolved Ig only applying to light-chain MGUS. Patients risk for progression according to the NCI2019 model was classified as low (0–1 points), intermediate (2 points) or high ( ≥ 3 points) risk.

For analyzes of time-to-progression (TTP) from 3 years after initial diagnosis and beyond, and for analyzes of patients migrating into higher risk groups over time, risk categories were simplified across all three models due to low patient numbers. In the simplified models, high vs. low risk was defined as follows: 0 risk factor vs. ≥ 1 risk factor in the Mayo2005 model, 0–1 risk factor vs. ≥ 2 risk factors in the Sweden2014 model, and 0–1 points vs. ≥ 2 points in the NCI2019 model.

To further compare the longitudinal performance of the three MGUS risk models, we compared them with the PANGEA model, developed to longitudinally assess patients´ individual risk of progression. The PANGEA model [10] comprises MCP, sFLCr, creatinine, hemoglobin trajectory, age and, if available, BMPC. We used the individual predicted risk at 3 years as continuous predictor in our analysis, with hazard ratios giving effects per 10% increments.

Patient routine assessments were captured yearly (+/- 6 months) for the first 5 years and additionally after 8 years. Serum and 24 h urine samples and, if indicated, imaging or bone marrow diagnostics were retrieved from the electronic medical records. Patient´s risk categorization in each model was reassessed at every timepoint for which data was available. Variables from previous timepoints were not carried forward.

The primary aims of this study were to assess longitudinal distribution and applicability of the three established MGUS risk stratification models during continuous disease monitoring in clinical practice.

### Statistical methods

Alluvial plots were used to visualize changes in risk stratification in the three different MGUS risk models over time. TTP was defined as time from first confirmed diagnosis of MGUS to progression to either smoldering multiple myeloma (SMM) or MM. Death without prior progression was treated as competing risk.

Cause-specific Cox regression models were used to analyze the prognostic impact of risk stratification models at different timepoints and longitudinally using the counting process approach. In case of complete separation due to low event numbers, Cox regression with Firth’s correction was used. Cumulative incidence curves based on the Aalen-Johansen estimator were calculated. Gray’s non-parametric test accounting for competing events was used to compare Cumulative incidence curves. C-Index provides discrimination according to Harrell’s concordance index. Migration from low to high-risk categories was additionally analyzed as time-dependent factor in Cox regression.

In case of a low MCP component in serum electrophoresis, quantification of the MCP (in g/l) was declared as “not quantifiable” by the laboratory. For statistical analyzes, “not quantifiable” MCP was defined as MCP 2 g/l.

P values below 0.05 were considered statistically significant. Software R 4.3 was used for all analyzes (The R Foundation for Statistical Computing, Vienna, Austria).

## Results

### Baseline patient characteristics and follow up

Among 518 identified patients with MGUS, 427 patients with follow up information and a complete set of clinical parameters at diagnosis were included in this analysis. Median patient age at MGUS diagnosis was 62 years (range 18–96), 188/427 (44%) of patients were female. Median time from first evidence of monoclonal gammopathy to confirmed MGUS diagnosis was 1 month (range 0–121) with 371/427 (87%), 15/427 (4%), and 41/427 (10%) patients diagnosed within 0–6, 7–12, and >12 months. Baseline characteristics of the cohort are listed in Table 1. Of 129/427 (30%) patients without bone marrow diagnostics, 55/129 (43%) patients had low-risk MGUS according to the MAYO2005 model and did not require bone marrow diagnostics in accordance with IMWG criteria [11]. For 28/427 (7%) patients, imaging was not available at baseline. Follow up information at the three-year time point was available for 181 (42%) and at the five-year time point for 113 (26%) patients. Data for reassessment of the risk group after 1, 2, 3, 4, 5, and 8 years was available for 121, 103, 90, 71, 47, and 26 patients, respectively, and 183/427 (43%) patients hat at least one additional risk stratification with any model during follow up period. Forty-eight out of 427 (11%) patients progressed after a median follow up of 2.5 years (95% CI 1.86–3.24), with 29 and 19 progressing into SMM and MM, respectively. Progression to SMM/MM at 5 years in the overall cohort was 11.9% (95% CI 7.4–16.5).

**Table 1:** Baseline patient characteristics.

MGUS patients (*n* = 427)		*N* (%)
Age at diagnosis	Median in years [range]	62 [18–96]
Sex	Female	188 (44.0)
	Male	239 (56.2)
Heavy chain isotype	IgG	319 (74.7)
	IgA	50 (11.7)
	IgM	30 (7.0)
	Biclonal	13 (3.0)
	Light chain	15 (3.5)
Light chain isotype	Kappa	244 (57.1)
	Lambda	169 (39.6)
	Biclonal	13 (3.0)
	Missing	1 (0.2)
MCP	Median in g/l [range]	3.75 [0.5–24.4]
	Not quantifiableª	174 (40.7)
	Missing	15 (3.5)
sFLCr	Median ratio [range]	1.67 [0.03–87.3]
BMPC	Median burden in % [range]	5.0 [1.0–9.5]
Immunoparesis	No	312 (73.1)
	Yes	95 (22.2)
	Missing	20 (4.7)

ªNot quantifiable was defined as MCP below the visually measurable size in serum electrophoresis.

### Longitudinal risk classification of MGUS

**Fig. 1:**
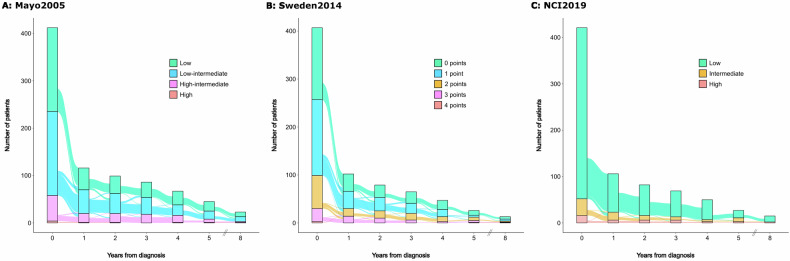
Alluvial plots illustrating the distribution and dynamic transition of patients across different risk groups over time in the three MGUS models. Alluvial plots for **A** the Mayo2005 model, **B** the Sweden2014 model, **C** the NCI2019 model.

Patient risk classification over time is visualized in Fig. 1. Considering all patients for whom at least one follow up was available in the Mayo2005 model, 40/183 patients (22%) migrated to a higher risk group, 20/183 patients (11%) migrated to a lower risk group, and 9/183 patients (5%) migrated between different risk groups, while the majority remained at their baseline risk group (114/183 patients [62%]). The proportion of low risk patients among all available patients at each subsequent timepoint remained stable: 177/412 (43%) at baseline, 46/116 (40%) at year 1, 37/99 (37%) at year 2, 32/86 (37%) at year 3, 29/67 (43%) at year 4, 20/45 (44%) at year 5, and 10/23 (43%) at year 8 (Fig. 1A). A similar migration pattern (43/157 [27%] of the patients migrate to a higher, 18/157 [11%] to a lower, 10/157 [6%] switch several times, and 86/157 [55%] remain in their initial risk group), and stable distribution of risk groups at the different time points was observed in the Sweden2014 model (Fig. 1B). Migration between risk categories in the NCI2019 model was numerically less compared to the Mayo2005 and Sweden2014 models: 136/162 (84%) remained at their baseline risk group, while 21/162 patients (13%) migrated to a higher risk group and only a few (3/162 patients [2%]) migrated to a lower risk group or migrated several times (2/162 patients [1%]). The NCI2019 model had a high proportion of low risk patients at all timepoints (baseline 369/421 [88%], 1 year 83/106 [78%], 2 years 66/82 [80%], 3 years 56/69 [81%], 4 years 43/50 [86%], 5 years 16/27 [59%], 8 years 13/15 [87%]; Fig. 1C).

### Risk of progression over time applying three different MGUS risk models

**Fig. 2:**
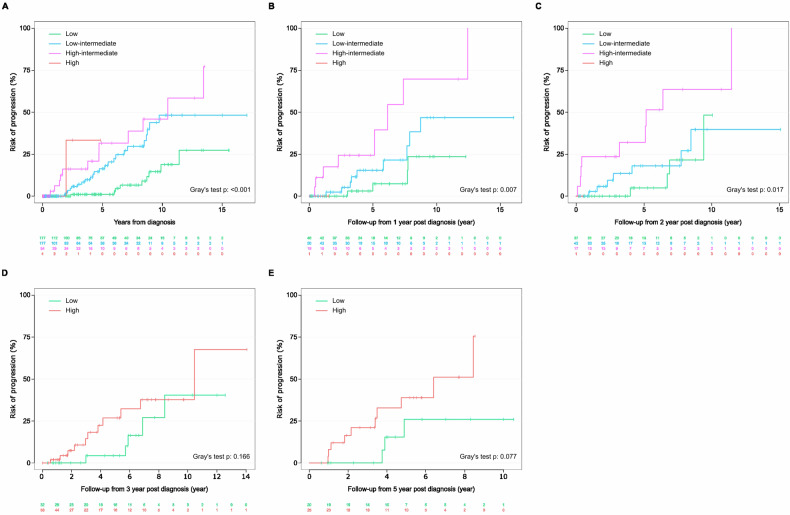
Risk stratification of patients over time according to the Mayo2005 risk model. Assessment of risk of progression **A** at initial diagnosis. Repeated evaluation of the individual risk at **B** 1 year, **C** 2 years, **D** 3 years using the simplified risk model, and **E** 5 years, using the simplified risk model.

**Fig. 3:**
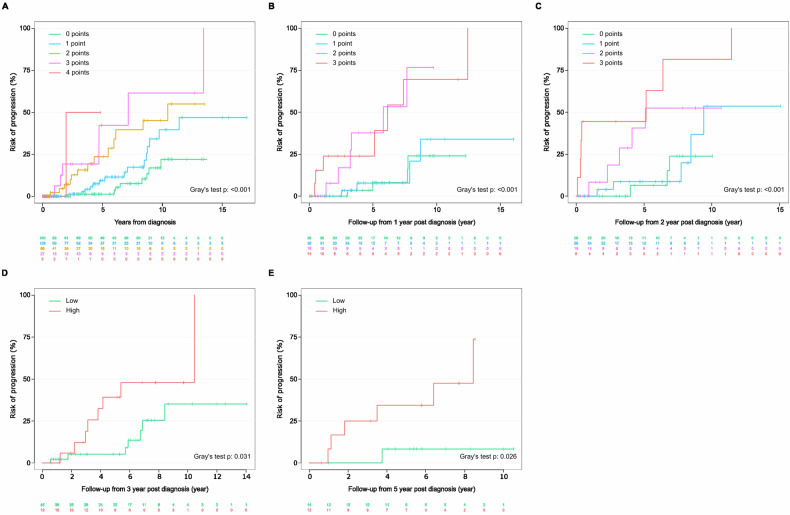
Risk stratification of patients over time according to the Sweden2014 risk model. Assessment of risk of progression **A** at initial diagnosis. Repeated evaluation of the individual risk at **B** 1 year, **C** 2 years, **D** 3 years using the simplified risk model, and **E** 5 years, using the simplified risk model.

**Fig. 4:**
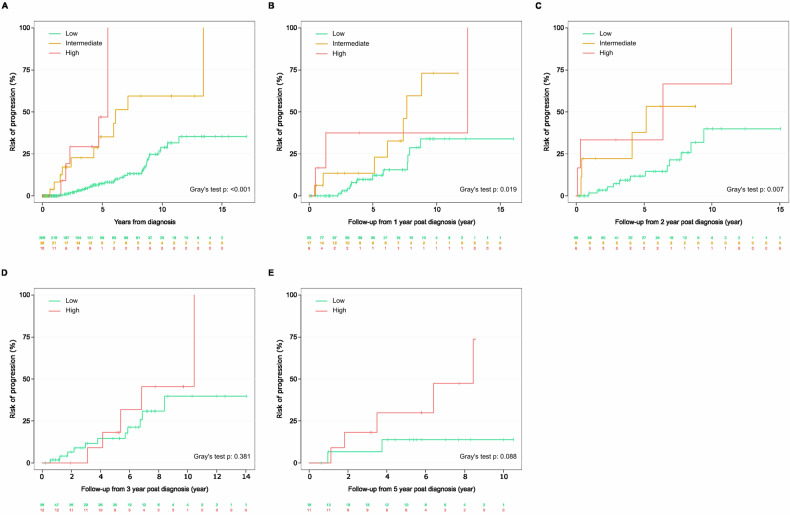
Risk stratification of patients over time according to the NCI2019 risk model. Assessment of risk of progression **A** at initial diagnosis. Repeated evaluation of the individual risk at **B** 1 year, **C** 2 years, **D** 3 years using the simplified risk model, and **E** 5 years, using the simplified risk model.

As previously described [7–9], all three established models discriminated patients in our cohort according to their TTP from initial diagnosis (Gray’s test *p* value < 0.001 for Mayo2005, Sweden2014, and NCI2019 models, respectively; Figs. 2A, 3A and 4A). Risk stratification from 1, 2, 3, and 5 years post diagnosis using the Mayo2005 model remained reliable (Gray’s test p value for TTP; for original model at year 1: 0.007, year 2: 0.017, and for a simplified model at year 3: 0.166, and year 5: 0.077; Fig. 2B–E). The Sweden2014 model similarly distinguished risk of progression upon yearly reassessment (Gray’s test p value for TTP; for original Sweden2014 model at year 1: <0.001, year 2: <0.001, and for a simplified model at year 3: 0.031, and year 5: 0.026; Fig. 3B–E). The NCI2019 model reliably differentiated risk groups over time (Gray’s test p value for TTP; for original NCI2019 model at year 1: 0.019, year 2: 0.007, and for a simplified model at year 3: 0.381, and year 5: 0.088; Fig. 4B–E). Further detailed results on the three MGUS risk scores 1 and 2 years post initial diagnosis are given in Supplemental Table 1.

To further evaluate the longitudinal applicability of the three established risk models in addition to the TTP analyzes, time-dependent Cox regression models were fitted (Table 2). Again, all three established models significantly differentiated patients regarding their risk of progression (*p* value < 0.001 for Mayo2005, Sweden2014, and NCI2019 models, respectively). Risk of progression over time gradually increased within higher risk groups in the respective risk models, for example in the NCI2019 model (intermediate vs. low risk: Hazard ratio [HR] = 4.82, 95% confidence interval [CI] 2.39–9.71, *p* < 0.001, and high vs. low risk: HR = 7.35, 95% CI 3.63–14.91, *p* < 0.001), with the exception of very small high risk groups, such as the baseline high risk groups in the Mayo2005 and Sweden2014 model (only 4 and 3 patients, respectively).

**Table 2:** Risk stratification at baseline and longitudinal risk stratification with all measurements used and evaluated as time-dependent covariates.

		Baseline risk stratification					Longitudinal risk stratification				
Risk model	Risk group	HR	95% CI	*p*-value	LRT *p*-valueª	C-index	HR	95% CI	*p*-value	LRT *p*-valueª	C-index
Mayo2005	Low	1.00			<0.001	0.732	1.00			<0.001	0.812
	Low-intermediate	3.70	1.79–8.27	<0.001			4.00	1.56–10.26	0.004		
	High-intermediate	5.72	2.46–13.73	<0.001			15.45	6.23–38.27	<0.001		
	High	18.20	1.91–82.87	0.018			5.03	0.59–43.10	0.141		
Sweden2014	0	1.00			<0.001	0.742	1.00			<0.001	0.831
	1	2.35	1.04–5,71	0.040			1.00	0.32–3.15	0.996		
	2	4.63	1.99–11.41	<0.001			4.98	2.02–12.23	<0.001		
	3	7.33	2.64–19.97	<0.001			17.34	7.08–42.49	<0.001		
	4	23.30	2.43–108.79	0.012			4.41	0.52–37.10	0.172		
NCI2019	Low	1.00			<0.001	0.669	1.00			<0.001	0.726
	Intermediate	3.85	1.87–7.40	<0.001			4.82	2.39–9.71	<0.001		
	High	9.21	3.17–22.77	<0.001			7.35	3.63–14.91	<0.001		
PANGEA	continuous^b^	1.19	1.10–1.28	<0.001	—	0.751	1.25	1.16–1.35	<0.001	—	0.814

ªLikelihood-ratio test (LRT).

^b^HR gives increase in risk per 10% increments.

In order to facilitate a comparison between initial and longitudinal risk stratification, both are presented in Table 2. As previously described, all three models stratify patients into appropriate risk groups, both initially and longitudinally. Nevertheless, when c-indices are compared, the longitudinal analysis reveals higher values than those observed in the risk assessment at baseline (e.g. in the Sweden2014 model c-index at baseline: 0.742 vs. c-index observed longitudinally: 0.831).

The PANGEA model revealed similar results with significant continuous increase in the risk of progression when applied longitudinally (HR = 1.25, 95% CI 1.16–1.35, *p* < 0.001), resulting in a c-index of 0.814 (vs. 0.751 at baseline; Table 2).

### Adverse impact of upstaging into higher risk categories over time

As described, small proportions of patients (25/183 [14%], 19/157 [12%] and 16/162 [10%] in the simplified Mayo2005, Sweden2014, and NCI2019 models, respectively) were assigned to a higher risk group over time. To assess whether upstaging to a higher risk category was associated with an increased risk of progression, we compared the migration to high risk categories with patients who remained stable in the low risk categories for all patients who were initially classified as low risk. In the three simplified risk stratification models, upstaging to a high-risk category was associated with increased risk of progression (Mayo2005 model: HR = 5.43, 95% CI 1.21–24.39, *p* = 0.027; Sweden2014 model: HR = 13.02, 95% CI 5.25–32.28, *p* < 0.001, and NCI2019 model: HR = 5.85, 95% CI 2.49–13.74, *p* < 0.001; Table 3).

**Table 3:** The impact of upstaging initial low risk in comparison to stable low risk patients.

Risk model	Risk group	HR	95% CI	*p*-value
Mayo2005	Low	1.00		
	Upstaging	5.43	1.21–24.39	0.027
Sweden2014	Low	1.00		
	Upstaging	13.02	5.25–32.28	<0.001
NCI2019	Low	1.00		
	Upstaging	5.85	2.49–13.74	<0.001

## Discussion

In this longitudinal analysis, contemporary MGUS risk scores reliably predicted risk of progression when applied serially post initial diagnosis. Furthermore, continuous assessment was superior to a single assessment at baseline. Assignment to a higher risk category was associated with an increased risk of progression during the course of surveillance.

Our study evaluated patients diagnosed with MGUS according to the latest IMWG2014 criteria and confirms that the three established risk models work in accordance with these criteria. Furthermore, light-chain MGUS is only explicitly included in the NCI2019 model. These patients are not considered in the other two models. Evaluation of the NCI2019 model in our cohort, including light-chain MGUS, demonstrates the prognostic value of the model across all disease subtypes.

Longitudinal risk assessment was already taken into account in the NCI2019 [9] and the PANGEA models [10]. The PANGEA model can be used for patients´ individual risk assessment at any time, regardless of the initial diagnosis. In contrast to the static models described in this retrospective study, it additionally incorporates creatinine concentration, age, BMPC burden (if available), and the hemoglobin trajectory. According to our results, disease risk can be updated over time with the repeated assessment of the common risk models, or the PANGEA model. Visram and colleagues evaluated commonly used risk stratification models in patients with SMM, namely the Mayo2018 and IMWG2020 risk models [12–14]. Similar to our current study in MGUS patients, SMM patients could reliably be stratified longitudinally and upstaging was associated with an increased risk of progression.

In the NCI2019 model, upstaging was associated with an increased risk of progression. Among patients who progressed in the NCI2019 study, the majority had high risk MGUS (53%). Of these high risk patients, 70% experienced upstaging to high risk from earlier low/intermediate risk [9]. Our current results are in line with this previous observation. In addition, the sFLCr criterion applied in the NCI2019 model is more narrow ( < 0.1 or > 10) as compared to the Mayo2005 model / normal sFLCr range ( < 0.26 or > 1.65). The altered sFLCr definition is associated with a high risk of progression [9] and likely excludes patients with abnormal sFLCr due to non-MGUS/MM renal damage [9, 15, 16]. Results from the iStopMM study to define novel sFLCr ranges have recently been presented and demonstrate that sFLCr varies more widely as previously described [16].

This study is limited by its retrospective nature and the fact that it was conducted at a single academic center. Further, the follow up time is relatively short. This aspect and the expected low progression rate of MGUS led to a small number of events during the observation period. Though required by the IMWG 2014 diagnostic criteria, small proportions of patients in our cohort did not have bone marrow diagnostics though recommended (74/427 [17%]) and/or imaging (28/427 [7%]). This may lead to the inclusion of SMM/MM patients in the initial analyzes on the Mayo2005, Sweden2014 and NCI2019 models [7–9], based on the current definition of MGUS. However, in accordance with these previous studies, we decided to include these patients in our analysis to reflect clinical practice. Further, our study focused on MGUS patients progressing into SMM and MM and did not capture data of patients with lymphoma or AL amyloidosis and underlying MGUS.

In conclusion, we demonstrate that serial assessment of risk of progression in MGUS patients is superior to a single assessment at first diagnosis. Migration of MGUS patients to a higher risk group over time is associated with an increased risk of progression and should prompt closer monitoring and restaging (e.g. with bone marrow and imaging diagnostics).

### Supplementary information

Supplemental Table1

## Data Availability

Data from this retrospective study is not publicly available. For requests, please contact the corresponding author (Elias K. Mai; elias.mai@med.uni-heidelberg.de).
