# Supplementary material for: Longitudinal assessment of established risk stratification models in patients with monoclonal gammopathy of undetermined significance
Source: Blood Cancer J. 2024 Aug 27;14(1):148. doi: 10.1038/s41408-024-01126-3 (PMC11349746; doi:10.1038/s41408-024-01126-3)
Supplement: Supplementary file 1 — Supplemental Table1 [file 41408_2024_1126_MOESM1_ESM.docx]

**Supplemental Table 1. Restratification of the risk groups one and two years after initial diagnosis.**

| **Risk stratification 1 year post diagnosis** | | | | | **Risk stratification 2 years post diagnosis** | | | | |  |  |
| --- | --- | --- | --- | --- | --- | --- | --- | --- | --- | --- | --- |
| **Risk model** | **Risk group** | **HR** | **95% CI** | **p-value** | **LRT p-valueª** | **C-index** | **HR** | **95% CI** | **p-value** | **LRT p- valueª** | **C-index** |
| Mayo2005 | Low | 1.00 |  |  | 0.011 | 0.722 | 1.00 |  |  | 0.032 | 0.737 |
|  | Low-intermediate | 2.37 | 0.81-8.12 | 0.119 |  |  | 1.56 | 0.50-5.54 | 0.447 |  |  |
|  | High-intermediate | 6.05 | 1.96-21.25 | 0.002 |  |  | 4.39 | 1.47-15.22 | 0.008 |  |  |
|  | High | 25.48 | 0.18-297.61 | 0.139 |  |  | 17.80 | 0.13-204.22 | 0.174 |  |  |
| Sweden2014 | 0 | 1.00 |  |  | 0.002 | 0.755 | 1.00 |  |  | 0.005 | 0.774 |
|  | 1 | 1.28 | 0.33-4.99 | 0.710 |  |  | 1.37 | 0.36-5.94 | 0.640 |  |  |
|  | 2 | 5.82 | 1.74-21.27 | 0.005 |  |  | 3.09 | 0.82-13.37 | 0.095 |  |  |
|  | 3 | 5.73 | 1.76-20.60 | 0.004 |  |  | 8.07 | 2.29-34.05 | 0.001 |  |  |
| NCI2019 | Low | 1.00 |  |  | 0.038 | 0.646 | 1.00 |  |  | 0.023 | 0.671 |
|  | Intermediate | 2.69 | 1.03-6.58 | 0.044 |  |  | 3.03 | 0.91-8.44 | 0.070 |  |  |
|  | High | 4.01 | 0.89-13.04 | 0.067 |  |  | 4.38 | 1.25-12.62 | 0.023 |  |  |

ªLikelihood-ratio test (LRT)
